# Clustering subspecies of *Aeromonas salmonicida* using IS*630* typing

**DOI:** 10.1186/1471-2180-13-36

**Published:** 2013-02-13

**Authors:** Nicole Studer, Joachim Frey, Philippe Vanden Bergh

**Affiliations:** 1 Institute of Veterinary Bacteriology, University of Bern, Länggassstrasse 122, Bern, Switzerland

**Keywords:** *Aeromonas salmonicida*, HCN-IS*630*-RFLP, IS element, Subtyping, Tc1 Mariner transposon, Salmonidae, Pathoadaptation

## Abstract

**Background:**

The insertion element IS*630* found in *Aeromonas salmonicida* belongs to the IS*630*-Tc1-mariner superfamily of transposons. It is present in multiple copies and represents approximately half of the IS present in the genome of *A. salmonicida* subsp. *salmonicida* A449.

**Results:**

By using High Copy Number IS*630* Restriction Fragment Length Polymorphism (HCN-IS*630*-RFLP), strains of various subspecies of *Aeromonas salmonicida* showed conserved or clustering patterns, thus allowing their differentiation from each other. Fingerprints of *A. salmonicida* subsp. *salmonicida* showed the highest homogeneity while ‘atypical’ *A. salmonicida* strains were more heterogeneous. IS*630* typing also differentiated *A. salmonicida* from other *Aeromonas* species. The copy number of IS*630* in *Aeromonas salmonicida* ranges from 8 to 35 and is much lower in other *Aeromonas* species.

**Conclusions:**

HCN-IS*630*-RFLP is a powerful tool for subtyping of *A. salmonicida*. The high stability of IS*630* insertions in *A. salmonicida* subsp*. salmonicida* indicates that it might have played a role in pathoadaptation of *A. salmonicida* which has reached an optimal configuration in the highly virulent and specific fish pathogen *A. salmonicida* subsp. *salmonicida*.

## Background

*Aeromonas salmonicida* is one of the predominant bacterial species found in fish and water samples [1]. While some *Aeromonas* species are able to cause opportunistic disease in warm- and cold blooded vertebrates, *A. salmonicida* seems to be specific for fish. *Aeromonas salmonicida* subsp. *salmonicida* a specific primary pathogen of *Salmonidae* (salmon, trout and char) has been known for decades to cause furunculosis. This bacterial septicaemia has a significant economic impact on aquaculture operations as well as on the wild stock of salmonids and some other fish species [2]. Bergey’s Manual of Systematic Bacteriology recognizes five subspecies of *A. salmonicida*: *salmonicida*, *achromogenes*, *smithia*, *pectinolytica* and *masoucida*[3]. *Aeromonas salmonicida* subsp. *salmonicida* is referred to as typical *Aeromonas salmonicida* by reason that these strains are very homogeneous and considered to be clonal [4,5]. Clinical strains that cannot be assigned to any of the known subspecies are referred to as *A. salmonicida* ‘atypical’. In recent years, it has been recognized that ‘atypical’ strains cause diseases in *salmonidae* and other fish species that differ from furunculosis. Therefore their importance is being increasingly recognized. The most common clinical manifestation observed, following infections with such strains, is chronic skin ulceration [6]. Due to a convoluted history of nomenclature and taxonomy of *Aeromonas* sp., clear assignment of strains using currently available methods remains sometimes confusing and controversial which makes epidemiological studies difficult [7]. Intraspecies phenotypic variability also makes phenotypic identification challenging on the species level [8]. A variety of molecular genetic methods have been employed for genetic classification of Aeromonads including mol% G + C composition, DNA-DNA relatedness studies, restriction fragment length polymorphism, pulsed-field gel electrophoresis, plasmid analysis, ribotyping, multilocus sequence typing, PCR and more [3,5]. Combination of 16S rDNA-RFLP analysis and sequencing of the gene *rpoD* was proposed as a suitable approach for the correct assignment of *Aeromonas* strains [9]. Moreover, analyzing strains by matrix assisted laser desorption/ionization time-of-flight mass spectrometry (MALDI-TOF) with an extraction method revealed 100% genus-level accuracy and 91.4% accuracy at species level [10]. However, this method was not able to discriminate *A. salmonicida* at the subspecies level.

Currently, no molecular approach gives a clear genotypic distinction of strains among *A. salmonicida* species. For this reason we elaborated a molecular genetic technique to achieve an adequate subtyping of all *Aeromonas salmonicida* subspecies. This method, named High Copy Number IS-Element based Restriction Fragment Length Polymorphism (HCN-IS-RFLP), has been successfully applied in numerous epidemiological studies for other pathogenic bacteria [11-15].

## Results

### Optimization of HCN-IS*630*-RFLP conditions

**Figure 1:**
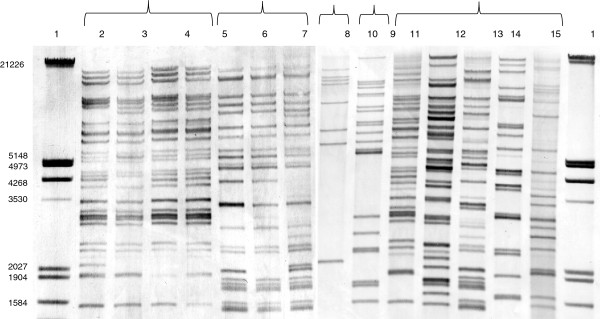
**Southern blot of *Xho*-I digested DNAs from different *A. salmonicida *strains hybridized with an IS*630*-specific probe.** Lanes 1 and 16, molecular size marker (sizes are indicated on the left in kilobase pairs); lanes 2 to 5 and 11, *A. salmonicida* subsp. *salmonicida* (JF2267, JF3224, JF3996, JF3507, JF3121 [formerly identified as atypical]); lanes 6 to 8 and 13, *A. salmonicida* subsp. *achromogenes* (JF3115, JF3116, JF2997, JF3123 [formerly identified as atypical]); lane 9, *A. salmonicida* subsp. *pectinolytica* (JF3120); lane 10, *A. salmonicida* subsp*. masoucida* (JF3118); lanes 12, 14 and 15, *A. salmonicida atypical* (JF3122, JF3124, JF3125).

IS*630* was selected because it is the IS element with the highest copy number in the genome of *A. salmonicida*[16]. Primers internal to the highly conserved IS*630* genes [GenBank: ABO88357.1] were designed to generate a probe on an intact IS fragment from the *A. salmonicida* subsp. *salmonicida* JF2267 genome. To obtain the most distinct banding pattern, the digestion by several restriction enzymes on a set of sequenced genomes (*A. salmonicida* subsp. *salmonicida* A449, *A. hydrophila* ATCC7966 and *A. veronii* B565) was predicted by computer analysis. XhoI that does not cut within our probe for IS*630* revealed a good resolution with a clear banding pattern and was therefore selected. A size window of 1375 bp to 21226 bp was defined on all southern blots as some hybridizing patterns with very large or small fragments were not sufficiently resolved (Figure 1). The genomic DNA sequence of *A. salmonicida* strain A449 [GenBank: CP000644.1] predicted that the probe would hybridize with 35 copies of IS*630* on XhoI fragments ranging from 1277 bp to 17948 bp (Additional file 1: Table S1).

**Figure 2:**
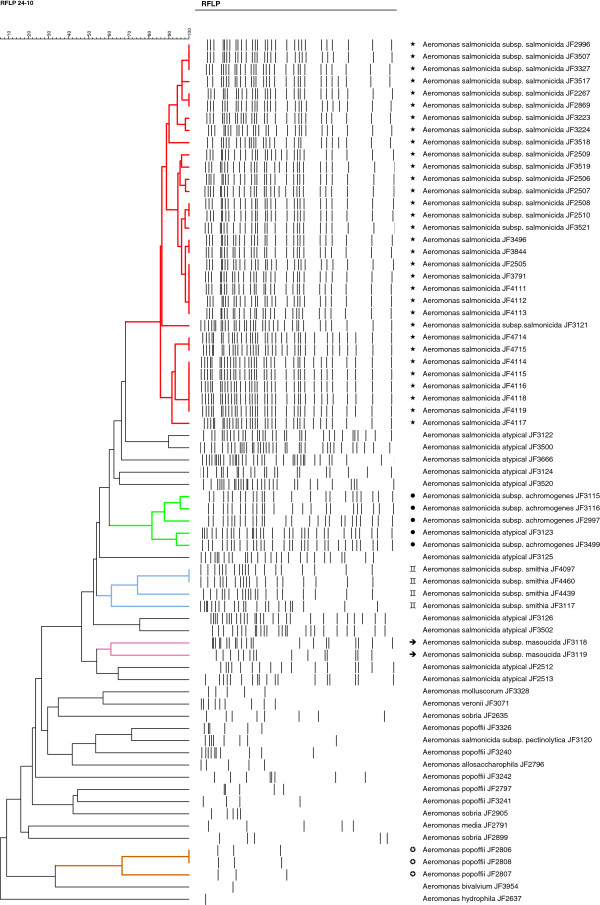
**Dendogram generated from the IS*630*-RFLP patterns of the 87 *Aeromonas *strains used in this study.** The tree was established by using the UPGMA clustering analysis with the BioNumerics software. In red (⋆), the *A. salmonicida* subsp. *salmonicida* cluster; in green (●), the *A. salmonicida* subsp. *achromogenes* cluster; in blue (), the *A. salmonicida* subsp. *smithia* cluster; in pink (➜), the *A. salmonicida* subsp. *masoucida* cluster; and in brown (**✪**), *A. popoffii* strains clustering together.

We analyzed the IS*630* RFLP-fingerprints of 87 *Aeromonas* sp. strains of various geographical origins, which comprised 31 *A. salmonicida* subsp. *salmonicida*, 4 subsp. *achromogenes*, 4 subsp. *smithia*, 2 subsp. *masoucida*, one subsp. *pectinolytica*, 12 *A. salmonicida* atypical strains, 8 *A. popoffii*, 5 *A. sobria* and *A. bestiarum*, 2 *A. hydrophila*, one *A. trota*, *A. enteropelogenes*, *A. simiae*, *A. eucrenophila*, *A. ichthiosmia*, *A. jandaei*, *A. molluscorum*, *A. bivalvium*, *A. allosaccharophila*, *A. media*, *A. veronii*, *A. caviae* and *A. culicicola* (Table 1). The fingerprints (Figure 1) of the analyzed strains were subjected to similarity analysis and are shown in Figure 2.

**Table 1:** ***Aeromonas *strains used in this study**

**JF N°**	**Synonyme**	**Species**	**Subspecies**	**Origin**	**Identified virulence characteristics**	**Pigment production (Day 6)**	**Ref**
JF2996	Austin98	*salmonicida*	*salmonicida*	Sediment in Riccarton Loch, Scotland	*ascV-, ascU-, aexT+, aopP+, aopO-, aopH+*	+++	[17,18]
JF3507	ATCC 33658 T, NCIMB 1102 T	*salmonicida*	*salmonicida*	*Salmo salar*, Scotland	*ascV-, aexT+, aopP+, aopO+, aopH+, acrD-*	+++	[18-20]
JF3327	F330/04	*salmonicida*	*salmonicida*	Arctic char, Switzerland, 2004	*ascV+, aexT+, aopP+, aopO+, aopH+*	++++	[18]
JF3517	4757	*salmonicida*	*salmonicida*	Turbot, Norway	*ascV+, aexT+, aopP+, aopO+, aopH+*	++++	[18]
JF2267	Fi 94 G	*salmonicida*	*salmonicida*	Arctic char, Switzerland, 1999	*ascV+, ascU+, aexT+, aopP+, aopO+, aopH+, acrD+*	+++	[17,18,20]
JF2869	CCUG 47405 (A)	*salmonicida*	*salmonicida*	Arctic char (*Savelinus alpinus*)	*aexT+, SacrD 3*^*′*^*+*	++++	-
JF3223	Fi 210	*salmonicida*	*salmonicida*	White fish, Switzerland, 1997	*ascV+, aexT+, aopP+, aopO+, aopH+*	++++	[18]
JF3224	R04/170	*salmonicida*	*salmonicida*	Brown trout, Switzerland, 2004	*ascV+, ascU+, aexT+, aopP+, aopO+, aopH+*	++++	[17,18]
JF3518	4704	*salmonicida*	*salmonicida*	Turbot, Norway	*ascV+, aexT+, aopP+, aopO+, aopH+*	++++	[18]
JF2509	CC72 - D640	*salmonicida*	*salmonicida*	Atlantic salmon, Canada, before 1960	*ascV+, aexT+, aopP+, aopO+, aopH+, acrD+*	++++	[18,20]
JF3519	3294	*salmonicida*	*salmonicida*	Arctic char, Switzerland, 1986	*ascV-, aexT+, aopP+, aopO+, aopH-*	++++	[18]
JF2506	CC 27–80/9-1	*salmonicida*	*salmonicida*	Atlantic salmon Norway	*ascV+, aexT+, aopP+, aopO+, aopH+, acrD+*	++++	[18,20]
JF2507	CC 29 - 74/2	*salmonicida*	*salmonicida*	Atlantic salmon, Scotland	*ascV+, aexT+, aopP+, aopO+, aopH+, acrD+*	++++	[18,20]
JF2508	CC 63- D-615	*salmonicida*	*salmonicida*	Atlantic salmon, Canada	*ascV+, aexT+, aopP+, aopO+, aopH+, acrD+*	++++	[18,20]
JF2510	CC 23/8019-5	*salmonicida*	*salmonicida*	Atlantic salmon Norway	*ascV+, aexT+, aopP+, aopO+, aopH+, acrD+*	++++	[18,20]
JF3521	2265	*salmonicida*	*salmonicida*	Wild atlantic salmon, Norway 1991	*ascV-, aexT+, aopP+, aopO+, aopH-*	++++	[18]
JF3496	F05/160	*salmonicida*	*salmonicida*	Wild brown trout, Switzerland, 2005	*ascV+, aexT+, aopP+, aopO+, aopH+*	+++	[18]
JF3844	F06/417	*salmonicida*	*salmonicida*	Arctic char, Switzerland, 2006	*ascV+, aexT+, aopP+, aopO+, aopH+*	+++	[18]
JF2505	MT 44/SS 10	*salmonicida*	-	non virulent for trout, Canada	A+, LPS+, *acrD-*	+++	[20]
JF3791	F06/385	*salmonicida*	-	Arctic char *Salvelinus alpinus*, Switzerland, 2006	*ascV+, aexT+, aopP-aopO+, aopH+*	+++	[18]
JF4111	F07/357(NiA)	*salmonicida*	-	*Salvelinus*, Switzerland, 2007	ND	+++	-
JF4112	F07/357 (NiB)	*salmonicida*	-	*Salvelinus*, Switzerland, 2007	ND	+++	-
JF4113	F07/357 (NiC)	*salmonicida*	-	*Salvelinus*, Switzerland, 2007	ND	+++	-
JF3121	As209	*salmonicida*	*salmonicida* [formerly atypical]	Wolf fish, UK	*ascV-, ascU-*	+++	[17]
JF4714	IMD1520	*salmonicida*	-	*Thymallus thymallus* (skin), Switzerland, 2009	ND	+++	-
JF4715	IMD 1521	*salmonicida*	-	*Thymallus thymallus* (kidney), Switzerland, 2009	ND	+++	-
JF4114	F07/357(LeA)	*salmonicida*	-	*Salvelinus* (liver), Switzerland, 2007	ND	+++	-
JF4115	F07/357 (LeB)	*salmonicida*	-	*Salvelinus* (liver), Switzerland, 2007	ND	+++	-
JF4116	F07/357 (LeC)	*salmonicida*	-	*Salvelinus* (liver), Switzerland, 2007	ND	+++	-
JF4118	F07/(MiB)	*salmonicida*	-	*Salvelinus* (kidney), Switzerland, 2007	ND	+++	-
JF4119	F07/357 (MiC)	*salmonicida*	-	*Salvelinus* (kidney), Switzerland, 2007	ND	+++	-
JF4117	F07/357 (MiA)	*salmonicida*	*salmonicida*	*Salvelinus* (spleen), Switzerland, 2007	ND	++++	-
JF3122	As204	*salmonicida*	atypical	Wrasse UK	*ascV+, aexT+, aopP + aopO-, aopH+*	++	[18]
JF3500	aAs 4143	*salmonicida*	atypical	Atlantic cod, Norway	*ascV+, aexT+, aopP + aopO+, aopH-*	++	[18]
JF3666	F06/211	*salmonicida*	atypical	Bleak (*Alburnus alburnus*), Switzerland, 2006	ascV+, aexT+, aopP- aopO-, aopH+	-	[18]
JF3124	As93	*salmonicida*	atypical	Plaice, Denmark	*ascV+, aexT+, aopP + aopO+, aopH+*	-	[18]
JF3520	4818	*salmonicida*	atypical	Atlantic Halibut, Norway, 2003	*ascV+, aexT-, aopP + aopO-, aopH+*	-	[18]
JF3115	ATCC 19261, NCIMB 1109	*salmonicida*	*achromogenes*	*Salmo trutta*	ND	+	-
JF3116	NCIMB 1110 T	*salmonicida*	*achromogenes*	Trout, Scotland	*ascV+, ascU+, aexT+, aopP+, aopO+, aopH+*	++	[17-19]
JF2997	F-265/87	*salmonicida*	*achromogenes*	Atlantic salmon, Iceland	*ascV+, ascU+, aexT+, aopP+, aopO+, aopH+*	++	[17,18]
JF3123	As183	*salmonicida*	*achromogenes* [formerly atypical]	Arctic char, Iceland	*ascV+, ascU+, aexT+, aopP+, aopO+, aopH+*	++	[17,18]
JF3499	aAs4101	*salmonicida*	*achromogenes*	Atlantic Cod, Iceland	*ascV+, aexT+, aopP + aopO+, aopH-*	-	[18]
JF3125	As 51	*salmonicida*	atypical	Rainbow trout, Norway	*ascV+, aexT+, aopP- aopO+, aopH+*	-	[18]
JF4097	-	*salmonicida*	*smithia*	*Salvelinus alpinus lepeschini*, Austria	*ascV+, aexT+, aopP+, aopO-, aopH+*	-	[21]
JF4460	-	*salmonicida*	*smithia*	*Salvelinus alpinus lepeschini*, Austria	*ascV-, aexT+, aopP+, aopO-, aopH+*	-	[21]
JF4439	-	*salmonicida*	*smithia*	*Salvelinus alpinus lepeschini*, Austria	*ascV+, aexT+, aopP+, aopO-, aopH+*	-	[21]
JF3117	NCMB13210, ATCC 49393	*salmonicida*	*smithia*	Roach, England	*ascV+, ascU+, aexT+, aopP-, aopO+, aopH+*	-	[17-19]
JF3126	As 54	*salmonicida*	atypical	Rainbow trout, Norway	*ascV-, aexT+, aopP-, aopO-, aopH-*	++	[18]
JF3502	aAs 4067	*salmonicida*	atypical	Spotted wolffish, Norway	*ascV+, aexT+, aopP+, aopO+, aopH+*	+	[18]
JF3118	ATCC 27013 T	*salmonicida*	*masoucida*	Salmon, Japan	*ascV+, ascU+, aexT+, aopP-, aopO-, aopH+*	-	[17-19]
JF3119	NCMB 2020	*salmonicida*	*masoucida*	same as ATCC 27013 (salmon, Japan)	ND	-	-
JF2512	CC 30/8038	*salmonicida*	atypical	Atlantic salmon, Canada, before 1960	*ascV+, ascU+, aexT+, aopP+, aopO+, aopH+, acrD+*	-	[17,18,20]
JF2513	CC 34/8030	*salmonicida*	atypical	Atlantic salmon, Canada, before 1960	*ascV+, ascU+, aexT+, aopP+, aopO+, aopH+, acrD+*	-	[17,18,20]
JF3328	848 T	*molluscorum*	-	Type strain	ND	-	[22]
JF3071	ATCC 51106, bg sobria HG8	*veronii*	-	?	ND	-	[19]
JF2635	429/01 # 1c; official JF2635	*sobria*	-	*Perca fluviatilis*, Switzerland, 2001	*ascV+, ascU+, acrD+*	-	[17]
JF3326	-	*popoffii*	-	Urinary tract infection, France	ND	-	[23]
JF3120	DSM 12609 T	*salmonicida*	*pectinolytica*	River water	*ascV-, aexT-, aopP-, aopO-, aopH-*	++++	[17,19]
JF3240	LMG 17542, IK-B-r-15-1	*popoffii*	-	Drinking water production plant, Belgium	ND	-	[24]
JF2796	CECT 4199	*allosaccharophila*	-	Type strain	ND	-	[19]
JF3242	LMG 17547, AG-9	*popoffii*	-	Drinking water treatment plant, Scotland	ND	-	[24]
JF2797	LMG 17541^T^, IK-0-a-10-3	*popoffii*	-	Drinking water production plant, Belgium	ND	-	[19,24]
JF3241	LMG 17544, IK-E-a- 14- 1	*popoffii*	-	Drinking water production plant, Belgium	ND	-	[24]
JF2905	Fi 125	*sobria*	-	Perch	*ascV+*	-	[25]
JF2791	ATCC 33907	*media*	-	Type strain NENT Nr. 2346-98	*ascV+, ascU+*	-	[17,19]
JF2899	F86/03-2	*sobria*	-	Perch	*ascV+*	-	[25]
JF2806	F533E	*popoffii*	-	Tap water, Switzerland, 2003	ND	-	[19]
JF2808	F600C	*popoffii*	-	Tap water, Switzerland, 2003	ND	-	[19]
JF2807	F548B	*popoffii*	-	Tap water, Switzerland, 2003	ND	-	[19]
JF 3954	868ET	*bivalvium*	-	Bivalve molluscs; Type strain	ND	-	[26]
JF2637	Fi 303	*hydrophila*	-	Ornamental fish	ND	-	-
JF2794	ATCC 49657, NENT Nr.2360-98	*trota *(*enteropelogenes*)	-	Human feces, India	ND	-	[19]
JF2785	CDC 9533-76	*bestiarum*	-	Type strain NENT Nr: N2341-98	ND	-	[19]
JF 4032	A28)A28B/1-1	*bestiarum*	-	Wild perch (*Perca fluviatilis*), Switzerland, 2007	ND	-	-
JF 4608	A28) 28B/1-1	*bestiarum*	-	Wild perch, Switzerland, 2009	*ascV+*	-	[22]
JF2804	F 530 D	*bestiarum*	-	Tap water	ND	-	-
JF3018	68	*bestiarum*	-	River water	ND	-	-
JF3070	S 6874 T	*simiae*	-	Type strain	ND	-	[19]
JF2786	ATCC 15468	*caviae*	-	Type strain NENT Nr. N2344-98	ND	-	[19]
JF2789	ATCC 7966	*hydrophila*	-	Type strain NENT Nr. : N2339-98	ND	-	[19]
JF2793	CIP 7433; ATCC 43979	*sobria*	-	Type strain NENT Nr.2352	ND	-	[19]
JF2929	Fi 179a	*sobria*	-	Perch, Switzerland	*ascV + SacrD+*	-	[22]
JF2788	NCMB 74; ATCC 23309	*eucrenophila*	-	Type strain NENT Nr. N2348-98	ND	-	[19]
JF3069	ATCC 49904 T	*ichthiosmia*	-	Type strain Antonella Demarta	ND	-	-
JF2790	ATCC 49568	*jandaei*	-	Type strain NENT Nr. 2355-98	ND	-	[19]
JF3067	CIP 107763 T	*culicicola*	-	Type strain	ND	-	[19]
JF3068	ATCC 49803 T	*enteropelogenes*	-	Type strain	ND	-	-

ND: not determined.

### HCN-IS*630*-RFLP profiles and stability of IS*630* insertions

A high degree of IS*630* polymorphism, both in a numerical and positional sense, was observed between the various *A. salmonicida* subspecies (Figure 1). However, the patterns revealed that IS*630* copy numbers and positions are well conserved within the given subspecies (Figure 1). The dendogram in Figure 2 is a RFLP tree that reveals the evolutionary relationship between strains analyzed. Strains of the subspecies *salmonicida*, *smithia*, *achromogenes* and *masoucida* each grouped together showing a similar banding pattern. The number of IS*630*-positive bands varied from 27 to 35 in *A. salmonicida* subsp. *salmonicida*, 23 to 33 in *achromogenes* and 19 to 21 in *smithia*. Within a subspecies, several bands were conserved: 21 in *salmonicida*, 20 in *achromogenes* and 13 in *smithia* subspecies. About 15 distinct patterns were observed in *A. salmonicida* subsp. *salmonicida* without showing geographical association. The IS*630* pattern of *A. salmonicida* subsp. *salmonicida* strain A449 as calculated from the genome sequence data closely clusters with these 15 patterns. In contrast, each pattern in the *achromogenes* cluster was different. In *A. salmonicida* subsp. *masoucida* 15 to 21 positive bands were detected and only 8 in the subspecies *pectinolytica*. Even though the copy numbers vary within the subspecies, the patterns form clusters for each subspecies. The most remarkable tight clustering was found for *A. salmonicida* subsp. *salmonicida*. This latter presents IS*630* patterns that only show minute differences among strains that were isolated from various continents and over a period of half a century. No pattern was specific of a given geographical region. The results showed also that strains JF3121 and JF3123, formerly classified as *A. salmonicida* atypical, clustered with *A. salmonicida* subsp. *salmonicida* (JF3121) and subsp. *achromogenes* (JF3123) (Figures 1 and 2) showing that they were misclassified previously.

The IS*630* pattern of *A. salmonicida* subsp. *salmonicida* strain JF 2267 that was subcultured for 4 days at 18°C and 25°C (in stressing conditions) to reach approximately 20 generations remained unchanged (results not shown) indicating a good stability of IS*630* under experimental growth conditions.

### Copy number of the IS*630* element and RFLP among other *Aeromonas* species

Other *Aeromonas* species revealed lower copy numbers of IS*630*: 5 in *A. molluscorum*, 5 to 8 in clinical *A. sobria strains*, 9 in *A. veronii*, 5 in *A. allosaccharophila* and *A. media*. Only one copy was found in *A. bivalvium* and a clinical strain of *A. hydrophila*. No signal for IS*630* was obtained in *A. caviae*, *A. trota*, *A. simiae*, *A. eucrenophila*, *A. ichthiosmia*, *A. jandaei*, *A. culicicola*, *A. enteropelogenes*, *A. bestiarum* and the type strains of *A. hydrophila and A. sobria*. Among the 8 strains of *A. popoffii* we found 6 very distinct patterns.

### Analysis of IS*630* abundance, localization and impact on the genome of *Aeromonas* species

**Figure 3:**
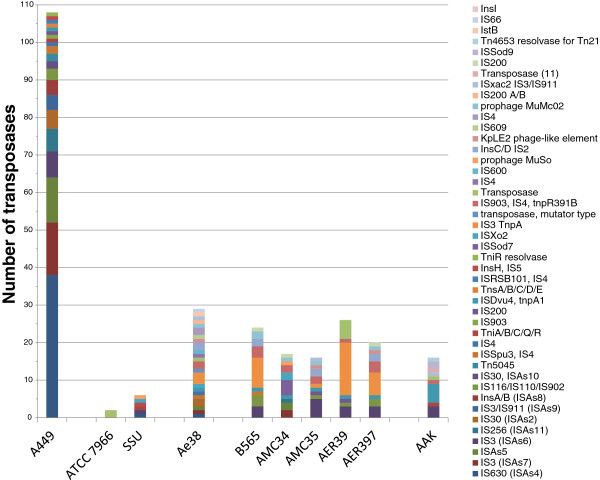
**Number of transposases and IS family affiliation in *Aeromonas *sp.***A. salmonicida* A449 [GenBank: CP000644.1, CP000645.1 and CP000646.1], *A. hydrophila* ATCC 7966 and SSU [GenBank: CP000462.1 and AGWR00000000.1], *A. caviae* Ae398 [GenBank: CACP00000000.1], *A. veronii* B565, AMC34, AMC35, AER39 and AER397 [GenBank: CP002607.1, AGWU00000000.1, AGWW00000000.1, AGWT00000000.1 and AGWV00000000.1], and *A. aquarorium* AAK1 [GenBank: AP012343.1].

**Figure 4:**
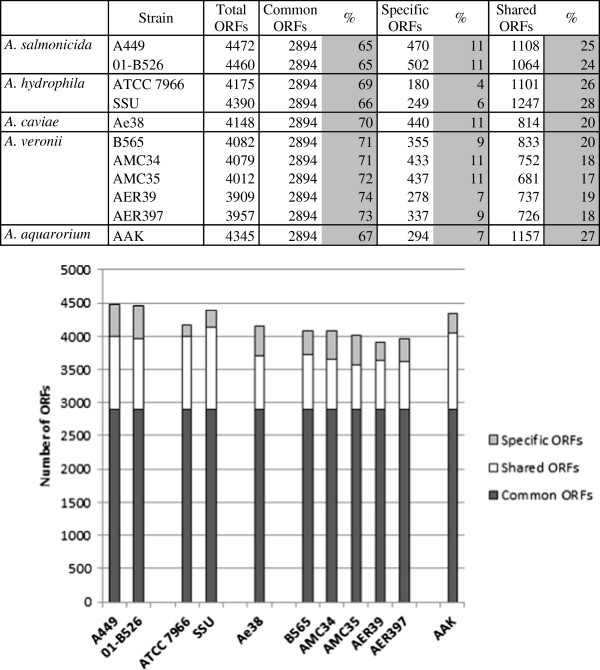
**Numerical comparison of common, shared and specific ORFs between several *Aeromonas *species.** The number of ORFs was calculated from Additional file 2: Table S2 without taking into account IS elements, tRNA and rRNA. In dark grey, the number of ORFs that are common among *Aeromonas* sp. In white, ORFs that are shared with at least one other *Aeromonas* species. In light grey, ORFs that are unique to the species. *A. salmonicida* subsp. *salmonicida* A449 and 01-B526, *A. hydrophila* ATCC 7966 and SSU, *A. caviae* Ae398, *A. veronii* B565, AMC34, AMC35, AER39 and AER397, and *A. aquarorium* AAK are illustrated in the graph.

In order to study the origin of IS*630* in *A. salmonicida*, we performed a profound analysis and comparison of published *Aeromonas* genomes (Additional file 2: Table S2). The genetic environment of IS*630* copies in the *A. salmonicida* subsp. *salmonicida* A449 genome is shown in detail in Additional file 1: Table S1. About 148 loci or DNA sequences forming 108 complete or partial IS units were found in the chromosome of *A. salmonicida* subsp. *salmonicida* A449 and on the plasmids pASA4/pASA5 [GenBank: CP000644.1, CP000645.1 and CP000646.1]. IS*630* (referred to as ISAs*4* in the Genbank genome annotation of *A. salmonicida* A449 and as ISAs7 in the corresponding manuscript [16]) was found to be present in 38 copies and was the most abundant family representing 35% of transposons in *A. salmonicida* A449 (Figure 3, Additional file 3: Table S3). The different copies are well-conserved and show 98% nucleotide sequences identity. The other 70 IS elements are ISAs7 (13%), ISAs5 (11%), ISAs6 (6%), ISAs11 (6%), ISAs2 (5%), ISAs9 (4%), ISAs8 (4%), and unclassified ISAs (16%) (Figure 3). 90% of the IS*630* copies reside in chromosomal regions that are specific to *A. salmonicida* subsp. *salmonicida* and were not found in other *Aeromonas*. Interestingly most of these loci correspond to known genes in bacterial genera other than *Aeromonas*. This is the case for instance for the hypothetical gene ASA_1385 (homology to VOA_002034 of *Vibrio* sp. RC586) that is directly linked to IS*630* in *A. salmonicida* subsp. *salmonicida* and is not found in other Aeromonads (Additional file 2: Table S2). In ISAs families other than IS*630*, 34 (31%) are directly adjacent to IS*630* showing that 66% of *A. salmonicida* A449 transposons are associated to genomic domains of variability. In comparison to other *Aeromonas* sp., *A. salmonicida* A449 contains 4 to 54 fold more transposases (Figure 3) which are not responsible for a genome-reductive evolution [27] because the total number of ORFs is stable in comparison to other Aeromonads (Figure 4). However they explain the high abundance of pseudogenes (170) in *A. salmonicida* subsp. *salmonicida*[16] in contrast to *A. hydrophila* ATCC 7966 which only contains 7 pseudogenes and 2 transposases.

## Discussion

HCN-IS*6110*-RFLP has been applied as a standard method to subtype *Mycobacterium tuberculosis* strains for years [28]. Moreover, RFLP based on IS elements has been employed to type numerous other pathogenic bacteria [14,15,29-31]. The published genome of *A. salmonicida* subsp. *salmonicida* A449 shows numerous IS elements among which 38 belong to the IS*630* family [GenBank: CP000644.1]. We therefore used HCN-IS*630*-RFLP as a new typing methodology for *Aeromonas* species.

IS*630* was present in different copy numbers and integrated at various sites between the different *A. salmonicida* subspecies. On the other hand banding patterns were conserved within subspecies (Figure 1). HCN-IS*630*-RFLP revealed that IS*630* is abundant in all subspecies of *A. salmonicida* allowing a good accuracy for genomic fingerprinting. Our results showed that RFLP profiles can be used to distinguish subspecies of *A. salmonicida* and to differentiate *A. salmonicida* from other *Aeromonas* species. They also indicate a high variability among strains of ‘atypical’ *A. salmonicida*. All strains of yet unclassified ‘atypical’ *A. salmonicida* consisted of a high number of IS*630* copies and were effectively related to the *A. salmonicida* cluster. Our method demonstrates that such ‘atypical’ strains represent a heterogeneous group that does not fit into the classification of the five described *A. salmonicida* subspecies. These strains might represent various subtypes of *A. salmonicida* subsp. *salmonicida* or novel subspecies of *A. salmonicida* that have adapted to particular ecological niches or respective hosts. On the other hand, all *A. salmonicida subsp. salmonicida* isolated since the 1950s and originating from all over the world have very similar patterns, indicating that they form a single clone showing pathoadaptational stability. Altogether, our results confirm those of a previous study comparing genomic profiles of clinical isolates of *Aeromonas salmonicida* using DNA microarrays [32]. With the origin and intensification of fish farming, genetic rearrangements occurring through IS transposition events could have been responsible for the selection and the emergence of this pathogenic fish specific clone. Such an adaptation process of a pathogenic bacterium towards its host was recently indicated in the *Mycoplasma mycoides* cluster for *Mycoplasma mycoides* subsp. *mycoides*[33]. Moreover, no unique pattern was associated to a specific geographical region of the world and we assume that this could be explained by the dissemination of *A. salmonicida* subsp. *salmonicida* strains between aquaculture countries via the intensification of the international trade in farmed salmon or by the natural migration of wild salmons.

Besides the epidemiologic and phylogenetic interests of IS*630* fingerprinting to subtype *A. salmonicida*, we studied the characteristics of this predominant IS element to reveal information concerning the pathoadaptation towards its specific host. Mobile genetic elements can exert different effects on bacterial genomes [11,34-36]. Through such genomic effects, IS*630* family has had an impact on the modulation of virulence genes in other bacteria [37-43]. In *A. salmonicida* 90% of the IS*630* copies reside in genomic regions that are variable between *Aeromonas* sp. (Additional file 1: Table S1) and 80% of these sites contain genes that are specific to *A. salmonicida* and are not encountered in other *Aeromonas* sp. suggesting that they constitute genomic islands. A part of these coding sequences are phages or hypothetical genes with homologues of characterized sequences in other environmental bacteria: i.e. the ‘Vibrio Seventh Pandemic cluster I’ (VSP-I), genes for the synthesis of polysaccharide capsule, lipopolysaccharide, S-layer, chitinase, cytolytic insecticidal delta-endotoxin, and some effectors (AopO and ApoH) of the type-three secretion system, the major virulence system of the bacterium. Based on these findings we assume that IS*630* elements could be used by environmental bacteria to exchange DNA fragments between each other by horizontal transfer. In the genomic islands where IS*630* is present, supplementary IS elements can be found, which might serve as hot spots for further insertions. This would allow the transposon and the genomic island to evolve with acquisition of new genes without disruption of existing loci. These observations could explain why the IS*630* elements remained stable within the *A. salmonicida* subsp. *salmonicida* genome.

Other interesting characteristics of IS elements homologous to IS*630* in *A. salmonicida* suggest that they could play a role in the co-adaptation of the bacterium with its host by trans-kingdom horizontal gene transfers through the bacterial T3SS: (i) such IS*630* elements are mostly present in Gram-negative bacteria that use a T3SS, (ii) their expression can be specifically induced or increased when bacteria are in direct contact with host cells [44] and (iii) several IS*630* are predicted to be T3SS effectors [45]. The Modlab^®^ T3SS effector prediction software gives for *A. salmonicida* IS*630* a positive output at 0.69 which means, that the IS*630* itself is a potential T3SS effector. Hence, when the bacteria colonize the host, the IS*630* expression could be induced and they could begin to exert their transposase activity by excising the transposon (composite if associated to adjacent additional DNA fragments) from the bacterial genome. Subsequently, the transposase linked to its transposon could be translocated into the host cell by the T3SS, reach the host genome in the nucleus, and finally perform its transposition.

Bacterial IS*630* elements constitute with the Tc*1*/mariner eukaryotic DNA transposon family, a superfamily [46]. It was demonstrated *in vitro* that eukaryotic members of this family are able to transpose into prokaryotic genomes [46]. We suppose that the opposite could also be possible as IS*630* itself could be translocated via type three secretion system from the pathogen to its host. In this perspective, our assumption could explain how the adaptive horizontal transfer of a bacterial mannanase gene (HhMAN1) into the genome of an invasive insect pest of coffee (*Hypothenemus hampei*) occurred in the immediate genetic vicinity of a Tc1/mariner transposon [47].

## Conclusions

In this study we describe HCN-IS*630*-RFLP as an adequate method for subtyping *A. salmonicida* strains and to differentiate *A. salmonicida* from other *Aeromonas* species. The high degree of conservation of HCN-IS*630*-RFLP profiles among strains of *A. salmonicida* subsp. *salmonicida* isolated from geographically most distant areas and over the period of half a century shows that practically all copies of IS*630* are stably integrated in this pathogen that has a well-defined host range. We therefore conclude that IS*630* might have contributed to the pathoadaptation of *A. salmonicida* to salmonidae and to the emergence of the subtype *A. salmonicida* subsp. *salmonicida*.

## Methods

### Bacterial strains and growth conditions

*Aeromonas* strains used in this study are listed in Table 1. Bacteria were grown on trypticase soy agar plates at 18°C for 3 to 6 days until sufficient bacteria were available for DNA extraction.

### Southern blot analysis with *A. salmonicida* subsp. *salmonicida* IS*630* probe

Total DNA extraction from each strain was performed with the Peqgold Bacterial DNA extraction Kit (Peqlab Biotechnologie, Erlangen, Germany). One microgram of DNA from each sample was digested overnight with XhoI restriction enzyme (Roche Diagnostics, Mannheim, Germany), loaded on a 0.7% agarose gel and subjected to electrophoresis for 4 to 5 hours. On each gel a DIG-labeled DNA Marker (Roche Diagnostics, Mannheim, Germany) and XhoI digested DNA from *A. salmonicida* subsp. *salmonicida* JF2267 were loaded for normalization. DNA bands were stained with ethidium bromide for control and transferred onto a nylon membrane (Roche Diagnostics, Mannheim, Germany) with a VacuGene apparatus (GE Healthcare Bio-Sciences). The IS*630* probe was prepared by PCR using primers Clust_asa1052_S6 (5^′^- AGGCAGAACTTGGGGTTCTT-3^′^) and Clust_asa1052_R4 (5^′^- ACAAAAGCGGGTTGTCACTC-3^′^) and DNA of *A. salmonicida* subsp. *salmonicida* JF2267 as a template. PCR was performed in 30 μL which contained 0.5 μL of Taq DNA polymerase (5 units/μL) (Roche Diagnostics, Mannheim, Germany), 300 nM of each primer, 1.75 mM MgCl_2_, 200 μM concentrations of each dNTP and 1 μl of the Digoxigenin-11-dUTP (1 nmol/μL) (Roche Diagnostics, Mannheim, Germany). Each reaction involved a denaturing step at 94°C for 5 min followed by 30 cycles of 10 sec at 94°C, 30 sec at 54°C and 60 sec at 72°C and a final extension step of 7 min at 72°C.

### Bioinformatic analysis

The hybridization patterns were scanned and the data were analyzed using the BioNumerics software version 6.6 (Applied Maths, Kortrijk, Belgium). Bands automatically assigned by the computer were checked visually and corrected manually. Cluster analysis of the IS-RFLP patterns was done by the unweighted pair group method with average linkages (UPGMA) using the Dice coefficient and the following parameters: 0.5% Optimization, 0% Band filtering, 0.5% Tolerance and ignore uncertain bands.

Prediction of T3SS effectors was performed with the Modlab^®^ online software (http://gecco.org.chemie.uni-frankfurt.de/T3SS_prediction/T3SS_prediction.html) [45].

### Stability of IS*630* in cultured *A. salmonicida* subsp. *salmonicida*

The stability of IS*630* under growth conditions in TSB medium was assessed by daily 100x dilution of a culture of strain JF2267 at 18°C and at 25°C during 4 days to reach 20 generations. Every day DNA was extracted from 10^9^ bacteria, digested with XhoI and submitted to southern blot hybridization.

## Abbreviations

HCN-IS-RFLP: High copy number insertion element restriction fragment length polymorphism; T3SS: Type-three secretion system; UPGMA: Unweighted pair group method with arithmetic mean.

## Competing interests

The authors have declared that no competing interests exist.

## Authors’ contributions

NS carried out the experiments, performed BioNumerics analysis and drafted the manuscript. JF participated in the coordination of the study and helped to draft the manuscript. PVB conceived of the study, participated in its design and coordination, carried out experiments, performed bioinformatic analysis and drafted the manuscript. All authors read and approved the final manuscript.

## Supplementary Material

Additional file 1: Table S1 Table showing for each *A. salmonicida* A449 IS*630* copy, the size of the XhoI-digested DNA fragment containing the IS, the inter- or intragenic localization, the characteristics of the adjacent genes, and the association to a region of variability or to other IS elements. Click here for file

Additional file 2: Table S2 Profound analysis and comparison of published *Aeromonas* genomes used for Figures 3 and 4. Grey: conserved ORFs; light green: ORFs specific of the species; yellow: IS*630*; pink: other IS elements; red: putative or characterized virulence factors; mauve: ORFs for resistance to antibiotic or heavy metal; dark green: ORFs associated to pili, fimbriae or flagella; blue: ORFs associated to phage; cyan: tRNA and rRNA; orange: ORFs with homology to eukaryotic genes. Click here for file

Additional file 3: Table S3 Detail of loci corresponding to transposons in *Aeromonas* sp. Click here for file

## References

[B1] Janda JM Abbott SL The genus *Aeromonas*: taxonomy, pathogenicity, and infection Clin Microbiol Rev 2010 23 35 73 10.1128/CMR.00039-09 20065325 PMC2806660

[B2] Hiney M Olivier G Woo PTK, Bruno DW Furunculosis (*Aeromonas salmonicidas*) Fish diseases and disordersviral, bacterial and fungal infections 1999 Walkingford, Oxfordshire, UK: CAB International 425 425 Volume 3.

[B3] Martin-Carnahan A Joseph SW Brenner DJ, Krieg NR, Staley JT, Garrity GM Family I. *Aeromonadaceae* Colwell, MacDonell and De Ley 1986, 474^VP^ Bergey’s Manual of systematic bacteriology, second edition, vol. 2 (The Proteobacteria), part B (The gammaproteobacteria) 2005 New York, NY: Springer 556 580

[B4] Wiklund T Dalsgaard I Occurrence and significance of atypical *Aeromonas salmonicida* in non-salmonid and salmonid fish species: a review Dis Aquat Organ 1998 32 49 69 9696626 10.3354/dao032049

[B5] Garcia JA Larsen JL Dalsgaard I Pedersen K **Pulsed-field gel electrophoriesis analyis of *****Aeromonas salmonicida***** ssp.***** salmonicida*** FEMS Microbiol Lett 2000 190 163 166 10981708 10.1111/j.1574-6968.2000.tb09280.x

[B6] Nilsson WB Gudkovs N Strom MS Atypical strains of *Aeromonas salmonicida* contain multiple copies of insertion element ISAsa4 useful as a genetic marker and a target for PCR assay Dis Aquat Organ 2006 70 209 217 16903232 10.3354/dao070209

[B7] Demarta A Tonolla M Caminada A Beretta M Peduzzi R Epidemiological relationships between *Aeromonas* strains isolated from symptomatic children and household environments as determined by ribotyping Eur J Epidemiol 2000 16 447 453 10.1023/A:1007675424848 10997832

[B8] Abbott SL Cheung WK Janda JM The genus *Aeromonas*: biochemical characteristics, atypical reactions, and phenotypic identification schemes J Clin Microbiol 2003 41 2348 2357 10.1128/JCM.41.6.2348-2357.2003 12791848 PMC156557

[B9] Beaz-Hidalgo R Alperi A Bujan N Romalde JL Figueras MJ Comparison of phenotypical and genetic identification of *Aeromonas* strains isolated from diseased fish Syst Appl Microbiol 2010 33 149 153 10.1016/j.syapm.2010.02.002 20227844

[B10] Lamy B Kodjo A Laurent F Identification of *Aeromonas* isolates by matrix-assisted laser desorption ionization time-of-flight mass spectrometry Diagn Microbiol Infect Dis 2011 71 1 5 10.1016/j.diagmicrobio.2011.04.014 21763094

[B11] McEvoy CR Falmer AA Gey van Pittius NC Victor TC van Helden PD Warren RM The role of IS*6110* in the evolution of *Mycobacterium tuberculosis* Tuberculosis (Edinb) 2007 87 393 404 10.1016/j.tube.2007.05.010 17627889

[B12] Thorne N Borrell S Evans J Magee J Garcia de Viedma D Bishop C Gonzalez-Martin J Gharbia S Arnold C IS*6110*-based global phylogeny of *Mycobacterium tuberculosis* Infect Genet Evol 2011 11 132 138 10.1016/j.meegid.2010.09.011 20920607

[B13] Bricker BJ Ewalt DR MacMillan AP Foster G Brew S Molecular characterization of *Brucella* strains isolated from marine mammals J Clin Microbiol 2000 38 1258 1262 10699036 10.1128/jcm.38.3.1258-1262.2000 PMC86392

[B14] Torrea G Chenal-Francisque V Leclercq A Carniel E Efficient tracing of global isolates of *Yersinia pestis* by restriction fragment length polymorphism analysis using three insertion sequences as probes J Clin Microbiol 2006 44 2084 2092 10.1128/JCM.02618-05 16757602 PMC1489393

[B15] Cheng X Nicolet J Poumarat F Regalla J Thiaucourt F Frey J **Insertion element IS1296 in *****Mycoplasma mycoides *****subsp. *****mycoides***** small colony identifies a European clonal line distinct from African and Australian strains.** Microbiology 1995 141 3221 3228 10.1099/13500872-141-12-3221 8574413

[B16] Reith ME Singh RK Curtis B Boyd JM Bouevitch A Kimball J Munholland J Murphy C Sarty D Williams J The genome of *Aeromonas salmonicida* subsp. *salmonicida* A449: insights into the evolution of a fish pathogen BMC Genomics 2008 9 427 10.1186/1471-2164-9-427 18801193 PMC2556355

[B17] Burr SE Pugovkin D Wahli T Segner H Frey J **Attenuated virulence of an *****Aeromonas salmonicida***** subsp.***** salmonicida***** type III secretion mutant in a rainbow trout model.** Microbiology 2005 151 2111 2118 10.1099/mic.0.27926-0 15942017

[B18] Burr SE Frey J Analysis of type III effector genes in typical and atypical *Aeromonas salmonicida* J Fish Dis 2007 30 711 714 10.1111/j.1365-2761.2007.00859.x 17958615

[B19] Küpfer M Kuhnert P Korczak BM Peduzzi R Demarta A Genetic relationships of *Aeromonas* strains inferred from 16S rRNA, *gyrB* and *rpoB* gene sequences Int J Syst Evol Microbiol 2006 56 2743 2751 10.1099/ijs.0.63650-0 17158971

[B20] Olivier G Moore AR Fildes J Toxicity of *Aeromonas salmonicida* cells to Atlantic salmon *Salmo salar* peritoneal macrophages Dev Comp Immunol 1992 16 49 61 10.1016/0145-305X(92)90051-D 1618355

[B21] Goldschmidt-Clermont E Hochwartner O Demarta A Caminada AP Frey J **Outbreaks of an ulcerative and haemorrhagic disease in Arctic char *****Salvelinus alpinus***** caused by***** Aeromonas salmonicida***** subsp.***** smithia.*** Dis Aquat Org 2009 86 81 86 19899353 10.3354/dao02110

[B22] Minana-Galbis D Farfan M Fuste MC Loren JG ***Aeromonas molluscorum *****sp. nov., isolated from bivalve molluscs.** Int J Syst Evol Microbiol 2004 54 2073 2078 10.1099/ijs.0.63202-0 15545437

[B23] Hua HT Bollet C Tercian S Drancourt M Raoult D *Aeromonas popoffii* urinary tract infection J Clin Microbiol 2004 42 5427 5428 10.1128/JCM.42.11.5427-5428.2004 15528763 PMC525237

[B24] Huys G Kampfer P Altwegg M Kersters I Lamb A Coopman R Luthy-Hottenstein J Vancanneyt M Janssen P Kersters K ***Aeromonas popoffii *****sp. nov., a mesophilic bacterium isolated from drinking water production plants and reservoirs.** Int J Syst Bacteriol 1997 47 1165 1171 10.1099/00207713-47-4-1165 9336924

[B25] Burr SE Goldschmidt-Clermont E Kuhnert P Frey J Heterogeneity of *Aeromonas* populations in wild and farmed perch, *Perca fluviatilis* L J Fish Dis 2012 35 607 613 10.1111/j.1365-2761.2012.01388.x 22620858

[B26] Minana-Galbis D Farfan M Fuste MC Loren JG ***Aeromonas bivalvium *****sp. nov., isolated from bivalve molluscs.** Int J Syst Evol Microbiol 2007 57 582 587 10.1099/ijs.0.64497-0 17329789

[B27] Song H Hwang J Yi H Ulrich RL Yu Y Nierman WC Kim HS The early stage of bacterial genome-reductive evolution in the host PLoS Pathog 2010 6 e1000922 10.1371/journal.ppat.1000922 20523904 PMC2877748

[B28] Alexander DC Guthrie JL Pyskir D Maki A Kurepina N Kreiswirth BN Chedore P Drews SJ Jamieson F *Mycobacterium tuberculosis* in Ontario, Canada: insights from IS*6110* restriction fragment length polymorphism and mycobacterial interspersed repetitive-unit-variable-number tandem-repeat genotyping J Clin Microbiol 2009 47 2651 2654 10.1128/JCM.01946-08 19494075 PMC2725669

[B29] Vergnes M Ginevra C Kay E Normand P Thioulouse J Jarraud S Maurin M Schneider D Insertion sequences as highly resolutive genomic markers for sequence type 1 *Legionella pneumophila* Paris J Clin Microbiol 2011 49 315 324 10.1128/JCM.01261-10 20980561 PMC3020482

[B30] Thomas R Johansson A Neeson B Isherwood K Sjostedt A Ellis J Titball RW Discrimination of human pathogenic subspecies of *Francisella tularensis* by using restriction fragment length polymorphism J Clin Microbiol 2003 41 50 57 10.1128/JCM.41.1.50-57.2003 12517824 PMC149632

[B31] Aebi M Bodmer M Frey J Pilo P Herd-specific strains of *Mycoplasma bovis* in outbreaks of mycoplasmal mastitis and pneumonia Vet Microbiol 2012 157 363 368 10.1016/j.vetmic.2012.01.006 22306036

[B32] Nash JH Findlay WA Luebbert CC Mykytczuk OL Foote SJ Taboada EN Carrillo CD Boyd JM Colquhoun DJ Reith ME Comparative genomics profiling of clinical isolates of *Aeromonas salmonicida* using DNA microarrays BMC Genomics 2006 7 43 10.1186/1471-2164-7-43 16522207 PMC1434746

[B33] Fischer A Shapiro B Muriuki C Heller M Schnee C Bongcam-Rudloff E Vilei EM Frey J Jores J The origin of the *‘Mycoplasma mycoides cluster’* coincides with domestication of ruminants PLoS One 2012 7 e36150 10.1371/journal.pone.0036150 22558362 PMC3338596

[B34] Mahillon J Chandler M Insertion sequences Microbiol Mol Biol Rev 1998 62 725 774 9729608 10.1128/mmbr.62.3.725-774.1998 PMC98933

[B35] Tanaka KH Dallaire-Dufresne S Daher RK Frenette M Charette SJ An insertion sequence-dependent plasmid rearrangement in *Aeromonas salmonicida* causes the loss of the type three secretion system PLoS One 2012 7 e33725 10.1371/journal.pone.0033725 22432045 PMC3303853

[B36] Muñoz-López M García-Pérez JL DNA transposons: nature and applications in genomics Curr Genomics 2010 11 115 128 10.2174/138920210790886871 20885819 PMC2874221

[B37] Houng HH Venkatesan MM Genetic analysis of *Shigella sonnei* form I antigen: identification of a novel IS*630* as an essential element for the form I antigen expression Microb Pathog 1998 25 165 173 10.1006/mpat.1998.0222 9817819

[B38] Larsson P Oyston PC Chain P Chu MC Duffield M Fuxelius HH Garcia E Halltorp G Johansson D Isherwood KE The complete genome sequence of *Francisella tularensis*, the causative agent of tularemia Nat Genet 2005 37 153 159 10.1038/ng1499 15640799

[B39] Sergeant M Baxter L Jarrett P Shaw E Ousley M Winstanley C Morgan JA Identification, typing, and insecticidal activity of *Xenorhabdus* isolates from entomopathogenic nematodes in United Kingdom soil and characterization of the *xpt* toxin loci Appl Environ Microbiol 2006 72 5895 5907 10.1128/AEM.00217-06 16957209 PMC1563616

[B40] Han HJ Kuwae A Abe A Arakawa Y Kamachi K Differential expression of type III effector BteA protein due to IS*481* insertion in *Bordetella pertussis* PLoS One 2011 6 e17797 10.1371/journal.pone.0017797 21423776 PMC3053399

[B41] Haneda T Okada N Nakazawa N Kawakami T Danbara H Complete DNA sequence and comparative analysis of the 50-kilobase virulence plasmid of *Salmonella enterica serovar Choleraesuis* Infect Immun 2001 69 2612 2620 10.1128/IAI.69.4.2612-2620.2001 11254626 PMC98198

[B42] Guiney DG Fierer J The role of the *spv* genes in *Salmonella pathogenesis* Front Microbiol 2011 2 129 21716657 10.3389/fmicb.2011.00129 PMC3117207

[B43] Stavrinides J Kirzinger MW Beasley FC Guttman DS E622, a miniature, virulence-associated mobile element J Bacteriol 2012 194 509 517 10.1128/JB.06211-11 22081398 PMC3256676

[B44] Münch A Stingl L Jung K Heermann R *Photorhabdus luminescens* genes induced upon insect infection BMC Genomics 2008 9 229 10.1186/1471-2164-9-229 18489737 PMC2422844

[B45] Lower M Schneider G Prediction of type III secretion signals in genomes of gram-negative bacteria PLoS One 2009 4 e5917 10.1371/journal.pone.0005917 19526054 PMC2690842

[B46] Plasterk RH Izsvák Z Ivics Z Resident aliens: the Tc1/mariner superfamily of transposable elements Trends Genet 1999 15 326 332 10.1016/S0168-9525(99)01777-1 10431195

[B47] Acuna R Padilla BE Flórez-Ramos CP Rubio JD Herrera JC Benavides P Lee SJ Yeats TH Egan AN Doyle JJ Adaptive horizontal transfer of a bacterial gene to an invasive insect pest of coffee Proc Natl Acad Sci USA 2012 109 4197 4202 22371593 10.1073/pnas.1121190109 PMC3306691

