# Supplementary material for: Clustering subspecies of Aeromonas salmonicida using IS630 typing
Source: BMC Microbiol. 2013 Feb 13;13:36. doi: 10.1186/1471-2180-13-36 (PMC3608246; doi:10.1186/1471-2180-13-36)
Supplement: Additional file 1: Table S1 — Table showing for each A. salmonicida A449 IS630 copy, the size of the XhoI-digested DNA fragment containing the IS, the inter- or intragenic localization, the characteristics of the adjacent genes, and the association to a region of variability or to other IS elements. [file 1471-2180-13-36-S1.doc]

| A449  locus | Predicted  XhoI  fragment  (bp)* | Intact, interrupted or partial IS630 | Inter- or  intragenic | Adjacent genes | Region of variability between *Aeromonas* sp. | Gene(s) specific of *A. salmonicida* and most similar homologue(s) | Other IS elements in the region of variability |
| --- | --- | --- | --- | --- | --- | --- | --- |
| ASA_0160 | 7056 | Intact | Inter | ASA_0159: putative SAM-dependent methyltransferase  ASA_0162 & ASA_0163: lipopolysaccharide O-Ag biosynthesis protein FlmB & FlmA | ASA_0137 to ASA_0164 | ASA_0161: methyl transferase  (BURPS1106A_3963, Burkholderia pseudomallei 1106a) | ASA_0144 & ASA_0145  (InsB & InsA, ISAS8) |
| ASA_0450 | 14212 | Interrupted by IS3 (ASA_0451 & ASA_0452) | Inter | ASA_0449: phage integrase  ASA_0453: SMI1 / KNR4 family (SUKH-1) | ASA_0443 (tRNA-Asn) to ASA_0457 (tRNA-Leu) | ASA_0453: SMI1 / KNR4 family (SUKH-1)  (ZP_04081902.1, Bacillus thuringiensis serovar pulsiensis BGSC 4CC1) | ASA_0451 & ASA_0452  (IS3 family)  ASA_0455 ASA_0456 |
| ASA_0524 | 5327 | Intact | Inter | ASA_0523: outer membrane channel protein TolC  ASA_0525: ADP-ribose pyrophosphatase | No | No | - |
| ASA_1023 | 6405 | Intact | Inter | ASA_1022: alpha-beta superfamily hydrolase, patatin  ASA_1024 & ASA_1025: pyoverdine biosynthesis protein PvcA & PvcB | ASA_1019 to ASA_1027 | No | - |
| ASA_1046 | 8072 | Interrupted by ISAS5 (ASA_1047 & ASA_1048) | Inter | ASA_1042 to ASA_1045: TniQ, TniB, TniA, TniC transposon  **ASA_1049 to ASA_1053: Vibrio Seventh Pandemic-I (VSP-I) cluster** | ASA_1034 to ASA_1054 | **ASA_1049 to ASA_1053: VSP-I cluster** | ASA_1042 to ASA_1045 (TniC, TniA, TniB, TniQ)  ASA_1047 & ASA_1048  (ISAS5) |
| ASA_1109 | 4555 | Intact | Inter | ASA_1108: small-conductance mechanosensitive ion channel  ASA_1110: MATE efflux family protein | No | No | - |
| ASA_1182 | 2307 | Intact | Inter | ASA_1181: autonomous glycyl radical cofactor  ASA_1183: uracil-DNA glycosylase | No | No | - |
| ASA_1304 | 2969 | Intact | Inter | ASA_1303: yfjL prophage protein  ASA_1305: hypothetical | ASA_1302 (tRNA –Ser) to ASA_1315 (tRNA-Thr) | ASA_1303: yfjL prophage protein  (PSYMO_03313, Pseudomonas syringae pv. mori str. 301020)  ASA_1305: hypothetical  (PcarbP_010200018474, Pectobacterium carotovorum subsp. brasiliensis PBR1692)  ASA_1306: hypothetical  (Dda3937_00827, Dickeya dadantii 3937)  ASA_1311: Rhs family protein  (Dda3937_04312, Dickeya dadantii 3937)  ASA_1312: hypothetical  (EpC_08520, Erwinia pyrifoliae Ep1/96)  ASA_1314: hypothetical  (no homologuous gene) | ASA_1307 & ASA_1308  (ISAS9)  ASA_1309 & ASA_1310  (ISAS5)  ASA_1313  (ISAS6, IS3 family) |
| ASA_1386 | 2431 | Intact | Inter | ASA_1385: hypothetical  ASA_1387: hypothetical | ASA_1383 to ASA_1388 | ASA_1384: hypothetical  (no homologous gene)  ASA_1385: hypothetical  (VOA_002034, Vibrio sp. RC586)  ASA_1387: hypothetical  (no homologous gene) | - |
| ASA_1400 | 3167 | Intact | Inter | ASA_1399a: putative acetyltransferase GNAT family  ASA_1401: IS3-family transposase, ISAS6 | ASA_1393 to ASA_1402 | ASA_1399a: putative acetyltransferase GNAT family  (V12B01_25509, Vibrio splendidus 12B01) | ASA_1401  (ISAS6, IS3 family) |
| ASA_1421 | 11004 | Intact | Inter | ASA_1420: NodT family efflux transporter, outer membrane factor (OMF) lipoprotein, outer membrane protein OprM  ASA_1422: dTDP-glucose-4,6-dehydratase | ASA_1419 to ASA_1459  (genes associated to LPS, polysacharidic capsule, and S-layer) | **ASA_1426 to ASA_1441: genes associated to tetragonal surface virulence array protein VapA**  **(homologous to Vibrio rotiferianus DAT722 genes)** | ASA_1454 & ASA_1455  (ISAS7, IS3 family) |
| ASA_1470 | 5117 | Intact | Intra | ASA_1469: dihydrolipoamide acetyltransferase | No | No | - |
| ASA_1616 | 9926 | Intact | Inter | ASA_1615: cysteinyl-tRNA synthetase  ASA_1617: hpothetical | ASA_1615 to ASA_1622 | ASA_1617: hypothetical  (ERFG_01251, Escherichia coli TW10509)  ASA_1618: hypothetical  (AlwoW_08768, Acinetobacter lwoffii)  ASA_1619: hypothetical  (ZP_00989641.1, Vibrio splendidus 12B01)  ASA_1620: hypothetical  (no homologous gene)  ASA_1621: diguanylate cyclase  (Varpa_3204, Variovorax paradoxus EPS) | - |
| ASA_1653 | 3931 | Intact | Intra | ASA_1652: ATPase involved in DNA repair | ASA_1649 to ASA_1655 | ASA_1652: ATPase involved in DNA repair  (VV2_1497, Vibrio vulnificus CMCP6) | - |
| ASA_1758 | 7112 | Intact | Inter | ASA_1757: hypothetical  ASA_1759: GGDEF/EAL protein | ASA_1756 to ASA_1759 | No | - |
| ASA_1780 | 1901 | Intact | Inter | ASA_1779: histone-like nucleoid structuring protein  ASA_1781: Na+/H+ antiporter | ASA_1779 to ASA_1781 | No | - |
| ASA_1872 | 9297 | Intact | Intra | ASA_1871: hypothetical | ASA_1870 to ASA_1873 | ASA_1871: hypothetical  (SeAg_B2099, Salmonella enterica subsp. enterica serovar Agona str. SL483) | - |
| ASA_1925 | 5633 | Intact | Inter | ASA_1924: GntR family transcriptional regulator  ASA_1926: glyoxalase/bleomycin resistance protein/dioxygenase | ASA_1920 to ASA_1930 | ASA_1921: SrpA, signal recognition particle RNA component  ASA_1923: hypothetical  (no homologous gene)  ASA_1924: GntR family transcriptional regulator  (SerAS12_1037, Serratia sp. AS12)  ASA_1928: SAM (and some other nucleotide) binding motif protein  (vfu_B00061, Vibrio furnissii NCTC 11218) | - |
| ASA_2141 | 7180 | Intact  + partial sequence (ASA_2114) | Inter | ASA_2138 to ASA_2140: ABC-type sugar transporter  **ASA_2142: chitinase** | ASA_2111 to ASA_2142 | ASA_2117 & ASA_2118: hypothetical  (DMR_16080 & DMR_16070, Desulfovibrio magneticus RS-1)  ASA_2119: integrase/recombinase  (EFW82225.1, Pseudomonas syringae pv. glycinea str. B076)  ASA_2121: DNA repair protein  (EHC04185.1, Shewanella baltica OS625)  ASA_2122: hypothetical  (Pgy4_27275, Pseudomonas syringae pv. glycinea str. race 4)  ASA_2123: hypothetical  (Rmet_3030, Cupriavidus metallidurans CH34)  ASA_2126 & ASA_2127: hypothetical  (no homologous genes)  **ASA_2128: cytolytic insecticidal delta-endotoxin**  **(CT1AA_BACTM, Bacillus thuringiensis)**  ASA_2131: transcriptional regulator, Crp/Fnr family  (MDS_2809, Pseudomonas mendocina NK-01) | ASA_2115 & ASA_2116  (ISAS7, IS3 family)  ASA_2124 & ASA_2125  (ISAS7, IS3 family)  ASA_2126a  (IS256)  ASA_2129 & ASA_2130  (ISAS7, IS3 family) |
| ASA_2237 | 2983 | Intact | Inter | ASA_2236: hypothetical  ASA_2238: hypothetical | ASA_2233 to ASA_2242 | ASA_2240: hypothetical  (FN3523_1005, Francisella cf. novicida 3523)  **ASA_2241: autolysin, phage lysozyme**  **(ZP_03216458.1, Salmonella enterica subsp. enterica serovar Virchow str. SL491)** | - |
| ASA_2252 | 6943 | Intact | Inter | ASA_2251: glycogen debranching enzyme, glgX  ASA_2253: hypothetical | ASA_2250 (tRNA-Met) to ASA_2253 | No | - |
| ASA_2478 | 17948 | Intact | Inter | ASA_2477: hypothetical  ASA_2479: hypothetical | ASA_2449 to ASA_2485 | ASA_2477, ASA_2479 & ASA_2480: hypothetical  (no homologous genes) | ASA_2454 & ASA_2455  (InsB & InsA, ISAS8)  ASA_2472  (IS903)  ASA_2481  (iso-IS1n protein insB)  ASA_2482  (ISAS6, IS3 family) |
| ASA_2512 | 3453 | Intact | Inter | ASA_2511: dipeptidase  ASA_2513: hypothetical | ASA_2510 to ASA_2516 | ASA_2513: hypothetical  (no homologous gene) | - |
| ASA_2838 | 7721 | Interrupted by ISAS5  (ASA_2839 & ASA_2840) | Intra | ASA_2837: putative non-ribosomal peptide synthetase | ASA_2830 to ASA_2842 | ASA_2833: 4'-phosphopantetheinyl tranferase  (P_01132582.1, Pseudoalteromonas tunicata D2)  ASA_2834: hypothetical  (PcitN1_03960, Pseudoalteromonas citrea)  ASA_2835: glycosyltransferase, family 4  (Bfae_12420, Brachybacterium faecium DSM 4810)  ASA_2836: non-ribosomal peptide synthase, NpnC  (AEU11003.1, Nostoc sp. 152)  ASA_2837: putative non-ribosomal peptide synthetase  (ZP_09674605.1, Paenibacillus dendritiformis C454)  ASA_2841: beta-N-acetylhexosaminidase  (NEMVEDRAFT_v1g201552, Nematostella vectensis) | ASA_2831 & ASA_2832  (ISAS7, IS3 family)  ASA_2839 & ASA_2840 (ISAS5) |
| ASA_2852 | 10597 | Intact | Inter | ASA_2851: alkyl hydroperoxide reductase subunit C  ASA_2853: LysR family transcriptional regulator | ASA_2850 to ASA_2853 | No | - |
| ASA_3034  &  ASA_ 3037 | 3050 | Intacts | Inter | ASA_3033, ASA_3035, ASA_3036: hypothetical  ASA_3038: threonine synthase , ThrC | ASA_3026 to ASA_3038 | ASA_3033, ASA_3035, ASA_3036: hypothetical  (no homologous genes) | ASA_3032  (IS200) |
| ASA_3080 | 15888 | Intact | Inter | ASA_3079: phage capsid scaffolding protein  ASA_3081: ISDvu4, transposase | ASA_3075 to ASA_3082 | ASA_3076: phage integrase  (EIQ03725.1, Shigella flexneri 2850-71)  ASA_3077: prophage regulatory protein  (YP_049728.1, Pectobacterium atrosepticum SCRI1043)  ASA_3079: phage capsid scaffolding protein  (ZP_01795108.1, Haemophilus influenzae PittII) | ASA_3081  (ISDvu4) |
| ASA_3458 | 1277 | Intact | Inter | ASA_3457: phage integrase  ASA_3459: hypothetical | ASA_3454 to ASA_3461 | ASA_3457: phage integrase  (EIW99631.1, Salmonella enterica subsp. enterica serovar Newport str. Levine 15)  ASA_3459: hypothetical  (CTS44_06248, Comamonas testosteroni S44) | - |
| ASA_3518 | 4390 | Intact | Inter | ASA_3517: aerotaxis receptor Aer  ASA_3519: hypothetical | ASA_3517 to ASA_3520 | ASA_3519: hypothetical  (no homologous gene) | - |
| ASA_3543 | 1538 | Intact | Inter | ASA_3542: hypothetical  ASA_3544: transcriptional regulator MerR family | ASA_3530 to ASA_3546 | ASA_3542: hypothetical  (no homologous gene) | - |
| ASA_3677 | 2848 | Intact | Inter | ASA_3676: hypothetical  ASA_3678: cyaY gene product | ASA_3663 to ASA_3678 | ASA_3664, ASA_3665, ASA_3667 to ASA_3669, ASA_3673 & ASA_3676: hypothetical  (no homologous genes)  ASA_3666: filamentation induced by cAMP protein fic  (EGH32378.1, Pseudomonas syringae pv. japonica str. M301072)  ASA_3670: phage regulatory protein  (PROSTU_00925, Providencia stuartii)  ASA_3671: mobilization protein  (ZP_07047705.1, Comamonas testosteroni S44)  ASA_3674: phage anti-repressor protein, AntA/AntB  (YPK_0617, Yersinia pseudotuberculosis YPIII)  ASA_3675: phage transcriptional regulator AlpA  (YPK_4164, Yersinia pseudotuberculosis YPIII) | - |
| ASA_3873 | 3426 | Intact | Inter | ASA_3872: phage integrase, prophage CP4-like integrase  **ASA_3874: CyaA, adenylate cyclase** | ASA_3827  (tRNA-Leu)  to ASA_3874 (prohage region) | Different genes of prophage origin | - |
| ASA_4327 | 6413 | Intact | Inter | ASA_4326: type I restriction-modification system M subunit  ASA_4328: hsdS, type I restriction-modification enzyme S subunit | ASA_4324 to ASA_4342 | ASA_4325: hypothetical  (ykris0001_9040, Yersinia kristensenii)  ASA_4326: type I restriction-modification system M subunit  (VIB_002047, Vibrio metschnikovii CIP 69.14)  ASA_4328: hsdS, type I restriction-modification enzyme S subunit  (EJA16672.1, Salmonella enterica subsp. enterica serovar Newport str. CVM 19449)  **ASA_4333: Predicted ATP-binding protein involved in virulence**  **(VIB_002045, Vibrio metschnikovii CIP 69.14)**  ASA_4334: hypothetical  (VIB_002044, Vibrio metschnikovii CIP 69.14) | ASA_4329 & ASA_4330  (ISAS5)  ASA_4331 & ASA_4332  (ISAS9)  ASA_4335,  ASA_4336, ASA_4339, ASA_4340,  ASA_4341  (TnsE, TnsD, TnsC, TnsB, TnsA, Tn7)  ASA_4337 & ASA_4338  (ISAS7) |
| ASA_P5G010 | 4170 | Intact | Inter | **ASA_P5G009: AopH T3SS effector**  ASA_P5G011: hypothetical | pASA5 | Several genes | ASA_P5G006  (IS256)  ASA_P5G007 |
| ASA_P5G093 & ASA_P5G0099 | 1930 | Intact & Partial | Inter | ASA_P5G092: transposase ISAS5  ASA_P5G094: fragment type II restriction enzyme, methylase subunit  **ASA_P5G098: AopO, serine/threonine protein kinase**  ASA_P5G100: transposase | pASA5 | Several genes | ASA_P5G090  (IS256)  ASA_P5G091 & ASA_P5G092  (ISAS5)  ASA_P5G100  ASA_P5G101  (IS30, ISAS10) |

*Predicted size of the XhoI digested fragment recognized by the IS*630* probe
